# Qwen TextCNN and BERT models for enhanced multilabel news classification in mobile apps

**DOI:** 10.1038/s41598-025-27497-6

**Published:** 2025-12-15

**Authors:** Dawei Yuan, Guojun Liang, Bin Liu, Suping Liu

**Affiliations:** 1 School of Computer Science, Guangdong University of Science and Technology, Dongguan, 523083 China; 2 Beijing Bitauto Information Technology Co., Ltd, Beijing, 100102 China; 3 School of Information Technology, Halmstad University, Halmstad, 30118 Sweden; 4 School of Computer Science and Technology, Jilin University, Changchun, 130012 China

**Keywords:** Multilabel classification, Large language models, BERT, TextCNN, Qwen, Computer science, Information technology

## Abstract

Mobile news classification systems face significant challenges due to their large scale and complexity. In this paper, we perform a comprehensive comparative study between traditional classification models, such as TextCNN and BERT based models and Large Language Models (LLMs), for the purpose of multi-label news categorization in mobile apps about the Chinese mobile news application. We evaluated the performance of conventional techniques, including a BERT model, along with Qwen models that have been tuned with instruction and fine-tuned using the LoRA technique, to optimize their effectiveness while preserving classification accuracy. Our experimental results show that BERT models perform best for multi-label classification with balanced datasets, while textCNN performs better for binary classification tasks. Our results also reveal that the LSTM and MLP classifiers consistently achieve the highest accuracy with text instruction prompts, while random embeddings achieve competitive accuracy. Furthermore, despite the low macro F1 scores due to class imbalance, consistent relative performance confirms the validity of our analysis. Our research reveals crucial information about the classification of automotive news, highlighting the importance of weighing technical prowess against deployment constraints when choosing model architectures.

## Introduction

With the rapid advancement of mobile Internet and content distribution platforms, the role of news classification in content distribution and recommendation systems becomes progressively significant. Mobile news classification systems face significant challenges due to their large scale and complexity. Recent studies show that mobile applications are highly dependent on machine learning techniques for information classification, requiring high accuracy and efficiency^1^. Modern news platforms use scalable text classification systems to provide personalized content for millions of users, while large-scale systems demonstrate real-time processing capabilities for massive data streams. However, deploying deep learning models on mobile devices presents substantial challenges, with studies showing higher failure rates as the complexity of the model increases^2^. Multilabel news classification systems typically manage 100 to 300 categories with varying complexity levels^3^, and news recommendation systems must handle diverse types of content while maintaining response times below 200 ms to keep users engaged^4^. Research indicates that lightweight convolutional neural networks provide a crucial balance between performance and resource efficiency for mobile deployment^5,6^. In the automotive industry, an accurate classification of news articles is essential for providing personalized content, improving user engagement, and effectively managing content. However, this domain faces several critical challenges that limit the effectiveness of current classification systems.

The first challenge lies in the complexity of multi-label classification, which poses considerable obstacles^7^. Traditional single-label classification techniques do not handle contemporary news articles that frequently cover multiple subjects simultaneously^8^. For example, an article might also discuss market trends, technological advancements, and government policies. Research indicates that the diversity of news content requires advanced classification methodologies to discern subtle distinctions between categories^9^, while unsupervised methods using models such as BERTopic illustrate the challenges of achieving accurate classification at both the overarching and detailed levels^10^. The second challenge is that severe class imbalance creates significant performance problems in real-world applications^11^. Some labels are prevalent, while others are rare, leading classification systems to favor common categories while underperforming on niche topics. The third challenge involves the constraints of mobile deployments, which limit the practical application of advanced models due to the limited computational resources and the strict latency requirements imposed to ensure a satisfactory user experience.

Extensive research has attempted to address these challenges. Traditional machine learning methods with custom features have been widely used^12,13^, but they have limited semantic understanding capabilities. Deep learning models such as TextCNN and RNN have improved feature extraction^14^, but still struggle with long-term dependencies and complex semantic relationships. Pre-trained models such as BERT have significantly improved semantic understanding, but face deployment challenges on mobile devices due to computational requirements. Large language models (LLMs) show promise for complex semantic tasks, but have problems with computational demands and response time^15^. To address these limitations, we performed a comprehensive comparison of traditional models and LLMs for multilabel automotive news classification. Our study develops a systematic framework to evaluate different model architectures for mobile deployment, considering both performance measures and practical constraints^16^. We created a large data set of more than 200,000 Chinese automotive news articles with 150 unique labels, providing a robust benchmark for multilabel classification evaluation. The primary contributions are as follows.

- We develop a comprehensive evaluation framework that combines theoretical performance metrics with the practical requirements of mobile deployment, such as latency and resource usage.
- We provide clear, data-driven model selection guidelines based on specific application needs, demonstrating the trade-offs between accuracy, speed, and deployment complexity for different model architectures.
- We present and validate optimization strategies for both traditional models and LLMs that enhance classification performance in mobile environments, particularly to address the challenge of severe class imbalance.

### Text convolutional neural network (TextCNN)

TextCNN represents an innovative leap in neural network applications for text classification. such as the methods introduced by Guo^17^ and Liu^18^, this model employs various convolutional layers with different kernel sizes to derive local text features on different scales. Its ability to detect critical n-gram patterns and local semantics through these convolutional methods contributes to its success^12,7^. For handling multi-label classification tasks, modifications have been made to TextCNN, incorporating multiple parallel output layers or sigmoid activation functions for simultaneous label prediction^19^. Despite its computational efficiency and generally favorable results^8^, TextCNN faces challenges with long-range dependencies and intricate contextual interactions^13^.

### Bidirectional encoder representations from transformers (BERT) models

The BERT models and their various adaptations have dramatically influenced text classification employing a strong pre-training paired with a fine-tuning strategy^20^. The bidirectional transformer structure improves comprehension of both context and semantic intricacies^21^. In Chinese text classification, custom versions such as *BERT-wwm*^22^ have emerged, adopting whole-word masking techniques to better accommodate the intricacies of the Chinese language. These models have delivered remarkable results in multi-label classification scenarios^16,23^, particularly in domain-specific applications^24^ and sentiment analysis^25^.

### Qwen models

The Qwen models signify a major advance in Chinese language processing, based on previous work on multilabel classification. These models incorporate innovative pre-training goals and structural improvements in transformer architecture. Developed by Alibaba, Qwen is proficient in understanding and producing Chinese language^26^, with models ranging from compact 0.5B parameters to expansive 7B and 14B configurations. The Qwen series features both foundational models and instruction-tuned versions, such as Qwen-Chat, which show improved results in a variety of NLP tasks. These models show exceptional abilities in multilabel classification challenges^27^, especially in managing specialized vocabulary and contextual comprehension^15,28^.

### Finetune and low-rank adaptation (LoRA) for Qwen

Recent advances in adaptation strategies for Large Language Models have resulted in improved solutions for text classification. A notable method is LoRA (Low-Rank Adaptation)^29^, recognized for its efficient fine-tuning of large models with minimal computational demands. This approach significantly reduces the number of parameters that need adjustment while preserving the performance of the model. In classification tasks, LoRA enables the adaptation of extensive models such as Qwen and its variations with reduced computational cost. This method shows great promise, especially in multilabel classification scenarios^30,31^, where conventional fine-tuning might be too expensive.

### Mobile APP news classifier

Classification systems for mobile news face unique challenges that require tailored solutions. They must find a balance between computational efficiency, accuracy, and resource management. Recent research emphasizes lightweight models that maintain classification accuracy within the constraints of mobile environments ^26^. Key optimization methods include model quantization, pruning, and architectural adjustments to reduce model size and accelerate inference. Hybrid strategies that integrate edge computing with device processing increase performance and resource management ^11^, frequently using batch processing and cache to improve operational efficiency. Class imbalance is a significant challenge in multilabel news classification.

## Methods

In this section, we describe the methodology for multi-label classification applied to news articles. For a summary $X = \{x_1, x_2, ..., x_n\}$ comprised of tokens $x_i$, our goal is to predict the label vector $Y = \{y_1, y_2,..., y_m\}$, where each $y_j \in \{0,1\}$ shows the applicability of the *j*-th label. We formulate a function $f: X \rightarrow Y$ that seeks to minimize classification errors while ensuring computational efficiency is upheld.

### TextCNN model training

We evaluated models that are of notable importance and popularity in the realm of Chinese news classification, specifically TextCNN, BERT, and Qwen models, examining their performance in binary and multi-label classification tasks. For multilabel classification, the TextCNN model handles input text with concurrent convolutional layers:

1 $$\begin{aligned} \begin{aligned} c_i&= \text {ReLU}(W_c \cdot x_{i:i+h-1} + b_c) \\ \hat{c}&= \text {MaxPool}([c_1, c_2, ..., c_{n-h+1}]) \\ y&= \sigma (W_o \cdot \hat{c} + b_o) \end{aligned} \end{aligned}$$

The size of the kernel is represented by *h*, with $W_c$ and $b_c$ linked to the convolution parameters, while $W_o$ and $b_o$ relate to the output layer parameters. Based on Kim’s study^32^, the model uses several kernel sizes, specifically 3, 4, and 5, to extract various n-gram features. For our approach, which is based on BERT, we employ the pre-trained Chinese BERT model together with a tailored classification head.

### BERT model training

2 $$\begin{aligned} \begin{aligned} H&= \text {BERT}(X) \\ h_{cls}&= H[0] \\ y&= \sigma (W_2 \cdot \text {ReLU}(W_1 \cdot h_{cls} + b_1) + b_2) \end{aligned} \end{aligned}$$

Here, *H* represents the contextual embeddings of BERT, while $h_{cls}$ signifies the embedding for the classification token. A multilayer perceptron that uses ReLU activation converts the BERT representation into label predictions, with $W_1$, $W_2$, $b_1$, and $b_2$ as adjustable parameters.

### Qwen model training

The instruction tuning accompanied by Low-Rank Adaptation (LoRA) is effective in fine-tuning these extensive language models for classification tasks.

3 $$\begin{aligned} \begin{aligned} W'&= W_0 + \Delta W \\ \Delta W&= BA \end{aligned} \end{aligned}$$

Here, $W_0 \in \mathbb {R}^{d \times k}$ represents the initial pre-trained weight matrix and $\Delta W$ is characterized by a low-rank decomposition into matrices $B \in \mathbb {R}^{d \times r}$ and $A \in \mathbb {R}^{r \times k}$. To address the classification task, we can treat it as a text generation problem using the following instruction: *Classify this news text into the appropriate category, Return only the category ID, *and then the model is trained to produce the appropriate label ID as:

4 $$\begin{aligned} P(Y|X) = \prod _{t=1}^{|Y|} P(y_t | y_{<t}, \text {Prompt}(X)) \end{aligned}$$

Here, $y_t$ denotes the token located at position *t* within the generated sequence, while $y_{<t}$ means all the tokens generated prior to position *t*.

### Loss function and training strategy

In the case of the TextCNN and BERT models, we employ a binary cross-entropy loss with applied weighting.

5 $$\begin{aligned} \mathscr {L} = -\frac{1}{m}\sum _{j=1}^m \omega _j(y_j\log (\hat{y_j}) + (1-y_j)\log (1-\hat{y_j})) \end{aligned}$$

where the weight $\omega _j$ connected to the label *j* is defined by:

6 $$\begin{aligned} \omega _j = \frac{N}{2N_j} \end{aligned}$$

Moreover, *N* signifies the total number of samples and $N_j$ indicates the number of samples related to the label *j*. This weighting method helps balance the impact of each label during training.

7 $$\begin{aligned} \mathscr {L}_{CLM} = -\sum _{t \in \text {label tokens}} \log P(y_t | y_{<t}, \text {Prompt}(X)) \end{aligned}$$

The loss for prompt tokens is masked to ensure that the model focuses on accurately predicting the correct sequence of labels.

**Algorithm 1:**
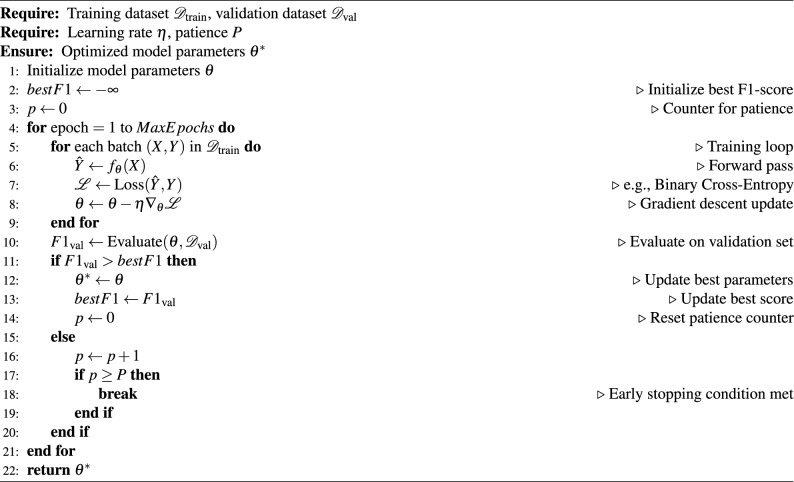
Standard model training.

**Algorithm 2:**
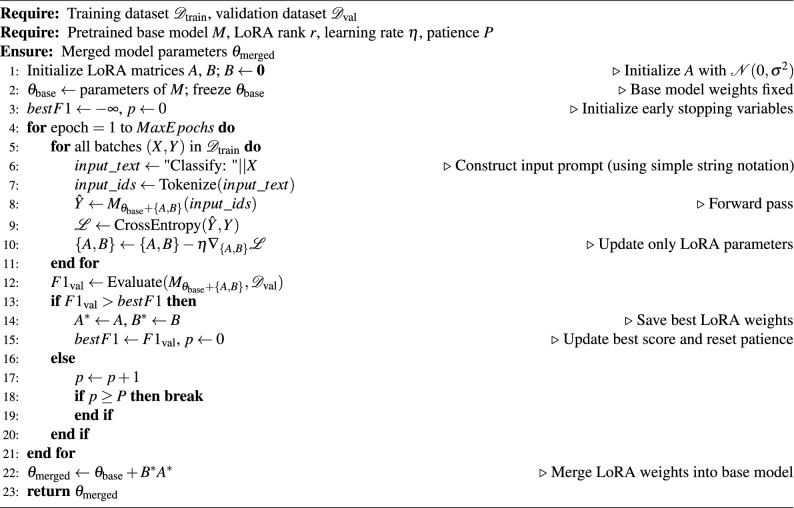
LoRA fine-tuning for Qwen models.

The algorithm 1 and 2 presents two different training methods. The standard approach employs weighted binary cross-entropy loss with early stopping based on validation F1 scores to prevent overfitting. The LoRA fine-tuning method provides a parameter-efficient alternative for large-language models like Qwen, where only low-rank adaptation matrices are trained while the base model remains frozen, which uses instruction-based prompts and masked loss calculation, significantly reducing computational requirements while maintaining performance.

## Results

### Research questions

We conducted comprehensive experiments to evaluate different model architectures and embedding strategies for multi-label news classification. Our research addresses four key questions:

- RQ1: How do TextCNN and BERT-based models perform when handling different numbers of labels in binary and multi-label scenarios?
- RQ2: How do various embedding strategies affect the performance of TextCNN and BERT-based models?
- RQ3: How effective are instruction-tuned large language models (LLMs), such as Qwen ^33^, for multi-label news classification tasks?
- RQ4: How do prompt engineering, resource constraints, and label filtering strategies affect the practical deployment and optimization of Qwen models for automotive news classification?

### Experimental setting

**Table 1:** Experimental configuration for multi-label news classification.

Parameter	Value
GPU	$2 \times \text {RTX } 4090$ ($24\text {GB}$)
CUDA/System RAM	$11.8 / 128\text {GB DDR}5$
Storage	$2\text {TB NVMe SSD}$
Framework	$\text {PyTorch } 2.1$
Source	Chinese mobile news app
Dataset size	$200\text {K}$ articles (8 : 2 split)
Labels	150 categories (multi-label)
Avg. length	$512 \pm 42$ tokens
Distribution	Long-tailed ($\alpha =1.25$)
TextCNN kernels	$\{7,8,9\}$
TextCNN filters	128
BERT base model	$\text {Chinese-BERT-base}$ ($110\text {M}$)
Qwen (LoRA)	$\text {Qwen-7B}$ ($\text {rank}=64$)
Batch size	32
Optimizer	$\text {AdamW}$ ($\beta _1=0.9, \beta _2=0.999$)
Learning rate	$2 \times 10^{-5}$ (cosine)
Max epochs	100 (Early Stop: 3)
Metrics	Micro $\text {F}1$, Macro $\text {F}1$
Inference hardware	Single $\text {RTX } 4090$
Inference batch	128
Precision	$\text {FP}16$

**Table 2:** Label distribution statistics in automotive news dataset.

Category	%	Category	%
New car analysis	14.81	Pre-sale	1.65
New car pricing	5.56	Configuration exposure	0.79
New car launch	5.45	Spy photos	0.51
Release appearance	3.04	Real vehicle exposure	0.45
Pre-heat	2.56	New car official images	0.44
		Declaration images	0.40
		New car arrival	0.13
Others: 83.22%			

Our approach utilized a 24GB RTX4090 GPU alongside automotive news datasets. Although the data set is accessible for those in research roles (contact us via email), company policy allows us to provide only testing subsets. The TextCNN framework adopts kernel sizes of 7, 8, and 9 to discern semantic differences in texts of various lengths, each size having 128 kernels to achieve a balance between feature extraction and computational efficiency. To select key features of each convolutional layer, we applied the k-max grouping with k = 1. We conducted our experiments using Python 3.9 and PyTorch 2.0, randomly splitting the dataset into 80% for training, 10% for validation and 10% for testing while preserving the original label ratios. A fixed random seed of 42 was set to ensure consistent results. Detailed configuration settings are presented in Table 1, and the distribution of labels is shown in Table 2.

### Evaluation metrics

To evaluate the performance of multilabel classification, we employ the conventional metrics used in label classification. For a test data set with *n* samples and *m* labels, let $Y_i = \{y_{i1}, ..., y_{im}\}$ be the true vector of labels and $\hat{Y}_i = \{\hat{y}_{i1}, ..., \hat{y}_{im}\}$ be the predicted vector of labels for sample *i* -th, where each element $y_{ij}, \hat{y}_{ij} \in \{0,1\}$. The following evaluation metrics are used:

Precision measures the proportion of correctly predicted positive labels among all predicted positive labels:

8 $$\begin{aligned} \text {Precision} = \frac{1}{n}\sum _{i=1}^n \frac{|Y_i \cap \hat{Y}_i|}{|\hat{Y}_i|} \end{aligned}$$

Recall represents the proportion of correctly predicted positive labels among all actual positive labels.

9 $$\begin{aligned} \text {Recall} = \frac{1}{n}\sum _{i=1}^n \frac{|Y_i \cap \hat{Y}_i|}{|Y_i|} \end{aligned}$$

The F1 score provides a balanced measure between precision and recall.

10 $$\begin{aligned} \text {F1-score} = \frac{2 \times \text {Precision} \times \text {Recall}}{\text {Precision} + \text {Recall}} \end{aligned}$$

Micro-averaging aggregates contributions from all classes, treating each instance equally to calculate the average metric.

11 $$\begin{aligned} \text {Micro-F1} = \frac{2 \times \text {Micro-P} \times \text {Micro-R}}{\text {Micro-P} + \text {Micro-R}} \end{aligned}$$

Macro-averaging computes the metric separately for each class and averages the results, giving equal importance to each class, independent of their support:

12 $$\begin{aligned} \text {Macro-F1} = \frac{1}{m}\sum _{j=1}^m \frac{2 \times P_j \times R_j}{P_j + R_j} \end{aligned}$$

where $P_j$ and $R_j$ represent the precision and recall of the *j*-th label, respectively. Micro-averaging emphasizes classes with more instances, making it suitable for imbalanced datasets. Macro-averaging treats all classes equally, providing overall performance insights across all labels regardless of their distribution.

### Experimental analysis

#### RQ1: How do TextCNN and BERT-based models perform when handling different numbers of labels in binary and multi-label scenarios?

**Fig. 1:**
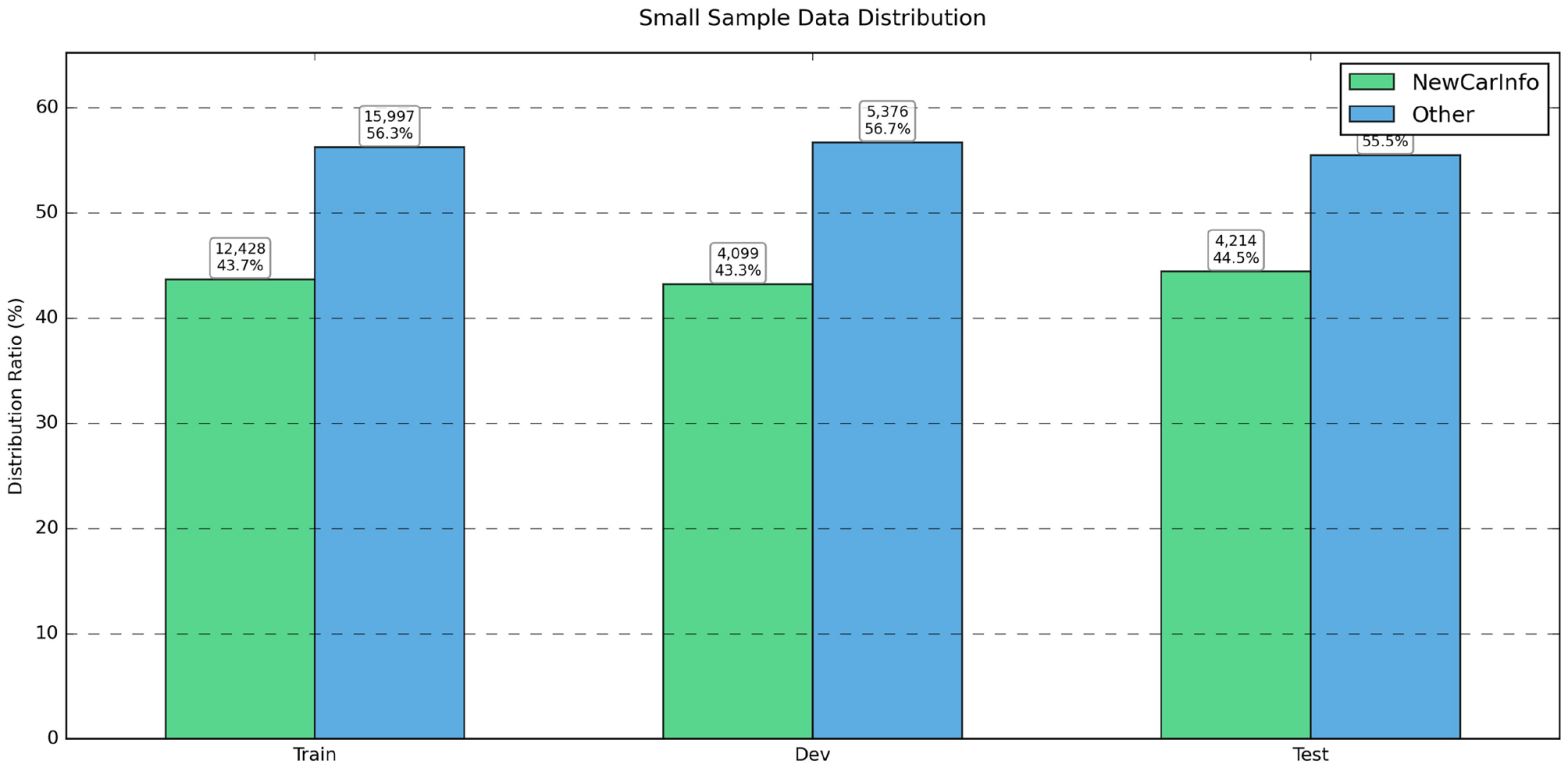
Small sample dataset distribution.

**Fig. 2:**
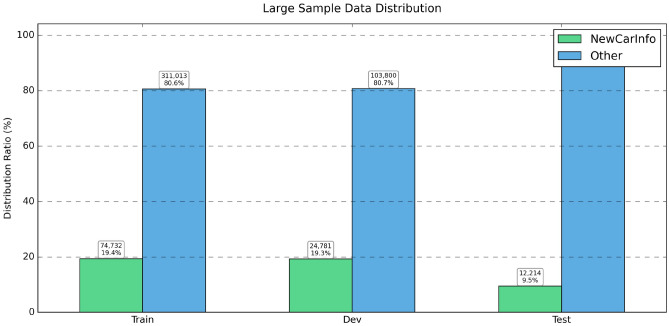
Large-scale dataset distribution.

**Fig. 3:**
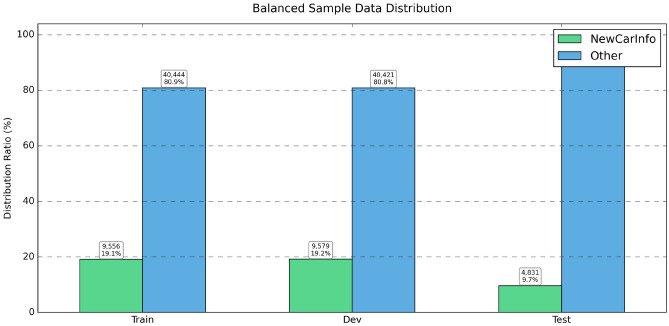
Balanced dataset distribution.

**Table 3:** Performance results on small sample dataset.

Model variant	Metrics		
	Prec	Rec	F1
TextCNN (Single-label)	0.738	0.681	0.708
TextCNN (Single, Char)	0.781	0.621	0.692
TextCNN (Binary)	0.795	0.906	0.847
TextCNN (Binary, Char)	0.785	0.890	0.834
TextCNN (Binary, Enh.)	0.845	0.860	0.852

**Table 4:** Performance results on large-scale dataset.

Category	Metrics		
	Prec	Rec	F1
New car information	0.895	0.962	0.927
Other	0.721	0.466	0.567
Accuracy	0.876		
Macro average	0.808	0.714	0.747
Weighted average	0.865	0.876	0.865

**Table 5:** Performance results on balanced dataset.

Evaluation metric	Value
Precision	0.131
Recall	0.006
F1-Score	0.011
Correct predictions	27
Total predictions	206
Actual NewCarInfo	4831

**Table 6:** Performance comparison with other category included (TextCNN vs. BERT).

Category	TextCNN			BERT		
	Prec.	Rec.	F1	Prec.	Rec.	F1
Other	0.900	0.945	0.922	0.000	0.000	0.000
Low-performance categories (F1 $\le$ *0.5)*						
Spy photos	0.000	0.000	0.000	0.640	0.404	0.496
New car pricing	0.648	0.451	0.532	0.625	0.250	0.357
New car launch	0.679	0.361	0.472	0.733	0.057	0.105
New car analysis	0.612	0.371	0.462	0.679	0.223	0.335
Failed predictions (F1 = 0.0)						
Release appearance	0.567	0.173	0.265	0.000	0.000	0.000
Pre-sale	0.647	0.103	0.177	0.000	0.000	0.000
Pre-heating	0.556	0.093	0.160	0.500	0.000	0.001
Configuration exposure	0.000	0.000	0.000	0.000	0.000	0.000

**Table 7:** Performance comparison with other category excluded (TextCNN vs. BERT).

Category	TextCNN			BERT		
	Prec.	Rec.	F1	Prec.	Rec.	F1
High-performance categories (F1 $\ge$ *0.7)*						
New car analysis	0.900	0.953	0.920	0.946	0.990	0.967
Spy photos	0.872	0.515	0.648	0.963	0.714	0.820
New car launch	0.825	0.633	0.717	0.936	0.603	0.734
Moderate-performance categories (0.5 $\le$ *F1* $\le$ *0.7)*						
New car pricing	0.800	0.685	0.738	0.933	0.583	0.718
Pre-heating	0.805	0.482	0.603	0.874	0.675	0.761
Release appearance	0.756	0.596	0.667	0.876	0.631	0.733
Low-performance categories (F1 $\ge$ *0.5)*						
Configuration exposure	0.344	0.089	0.142	0.575	0.615	0.594

We evaluated the performance of TextCNN and BERT models under various label complexities by systematically adjusting sample sizes and label distributions across three different experimental setups. Figure 1 shows small sample distributions, we observed a fairly balanced label distribution between *NewCarInfo* and *Other* categories (44% vs 56%), which presents an optimal environment for the evaluation of binary classification. Table 3 indicates that under these circumstances, TextCNN performs better in binary tasks compared to multilabel methods, with its enhanced binary version achieving the highest F1 score of 0.852. character encoding versions show varied results: single-label character encoding achieves higher precision (0.781) but lower recall (0.621). In contrast, analysis of large datasets (Fig. 2) reveals significant class imbalance issues, with the *Other* category comprising 80–90% samples. Table 4 shows that despite the imbalance, TextCNN delivers remarkable results on *NewCarInfo* (F1: 0.927) but struggles with the dominant *Other* class (F1: 0.567). The weighted average metrics (F1 score: 0.865) imply that the model adapts reasonably well to class imbalance when there is ample data. However, the balanced data set experiment (Fig. 3) reveals a significant performance drop due to forced balance. Table 5 reports notably poor results (F1: 0.011, Precision: 0.131, Recall: 0.006), with only 27 correct predictions of 206 for *4,831* actual *NewCarInfo* class, which suggests that naive balancing techniques can severely harm model performance. In multilabel classification analysis, the TextCNN and BERT models exhibit distinct behaviors. When the *Other* category is included (Table 6), TextCNN excels at handling this broad class (F1: 0.922), but struggles with more specific automotive labels. Conversely, BERT fails to predict the *Other* category (F1: 0.000) due to training exclusions, but performs better on specific categories like *Spy Photos* (F1: 0.496). Removing the *Other* category (Table 7) greatly improves the performance for both models, with BERT generally outperforming TextCNN in most categories. BERT excels in *New Car Analysis* (F1: 0.967) and *Spy Photos* (F1: 0.820), while TextCNN shows more consistent, but generally inferior performance.

**Table:** 

Answer to RQ1	
TextCNN excels in binary classification with balanced datasets, achieving a F1 score of 0.852. However, performance drops significantly with unbalanced datasets and balanced sampling strategies. BERT demonstrates superior multilabel performance, achieving F1 scores of up to 0.967 for car-related categories. The presence of the *Other* category significantly affects model performance, emphasizing the importance of careful label design in real world applications.	

#### RQ2: How do various embedding strategies affect the performance of TextCNN and BERT-based models?

**Fig. 4:**
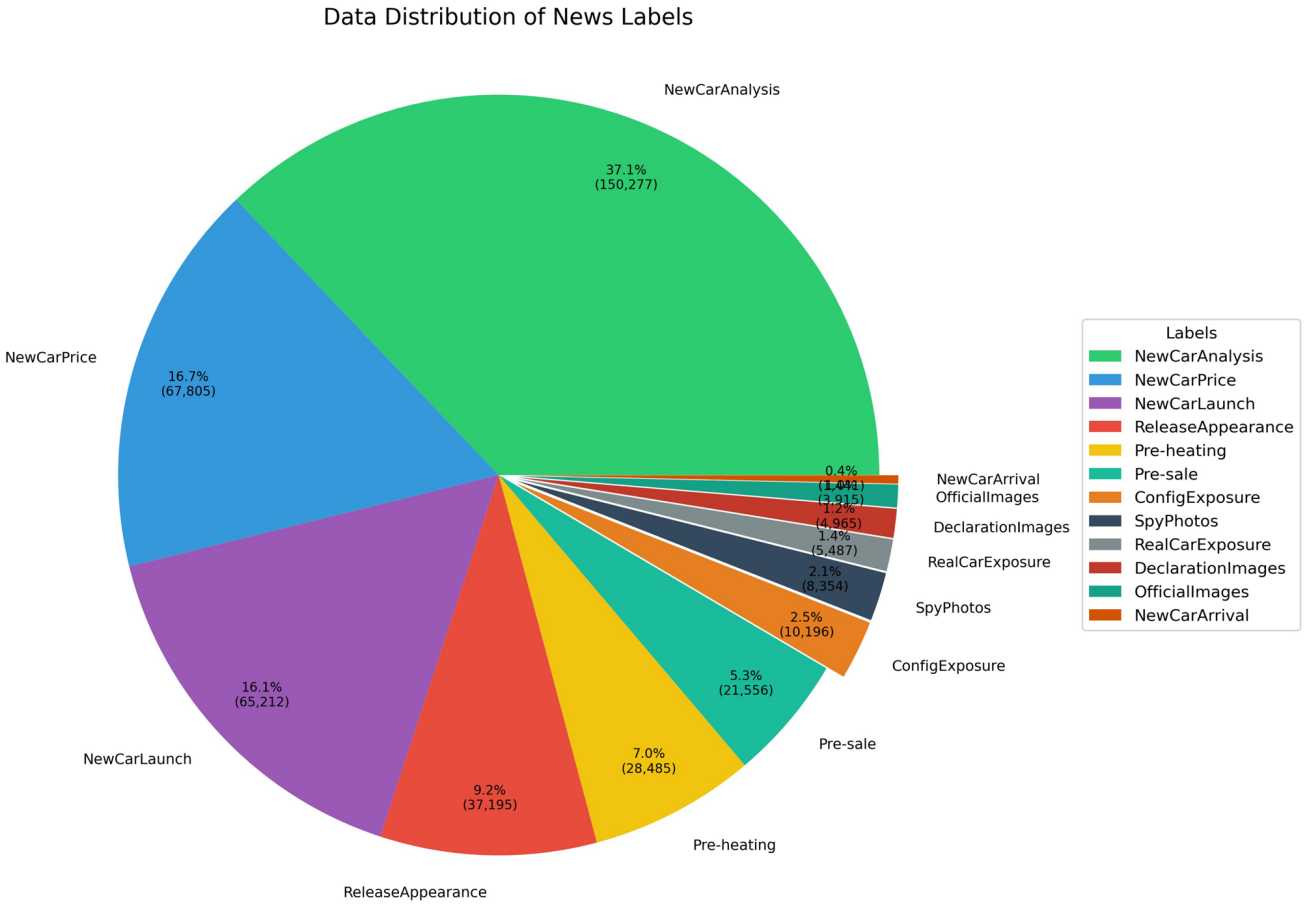
Data distribution of news labels.

**Table 8:** Comparative performance analysis of Chinese Pre-trained language models in automotive news classification.

Category	WWM BERT			BERT+TextCNN hybrid		
	Prec.	Rec.	F1	Prec.	Rec.	F1
Vehicle technical specifications						
New car analysis	0.995	0.980	0.988	–	–	–
Spy photos	0.927	0.981	0.953	–	–	–
Declaration images	0.929	0.963	0.946	–	–	–
New car pricing	0.935	0.903	0.919	–	–	–
Pre-sale	0.906	0.897	0.902	–	–	–
New car launch	0.932	0.866	0.898	–	–	–
Real car exposure	0.857	0.923	0.889	–	–	–
Configuration exposure	0.842	0.889	0.865	–	–	–
Official images	0.917	0.815	0.863	–	–	–
Pre-heating	0.902	0.798	0.847	–	–	–
Release appearance	0.871	0.802	0.835	–	–	–
New car arrival	0.727	0.800	0.762	–	–	–
Consumer guidance						
Vehicle brand analysis	–	–	–	0.969	0.963	0.966
Comparison guide	–	–	–	0.955	0.957	0.956
Single vehicle guide	–	–	–	0.911	0.927	0.919
Car purchase manual	–	–	–	0.939	0.900	0.919
Purchase techniques	–	–	–	0.939	0.859	0.897
Evaluation guide	–	–	–	0.904	0.906	0.905
Test drive	–	–	–	0.876	0.919	0.897
Multiple vehicle guide	–	–	–	0.928	0.852	0.888
Marketing guide	–	–	–	0.971	0.745	0.843
Vehicle showcase	–	–	–	0.886	0.795	0.838
Car sharing	–	–	–	0.945	0.729	0.823
Dealership pricing	–	–	–	0.829	0.782	0.805

**Fig. 5:**
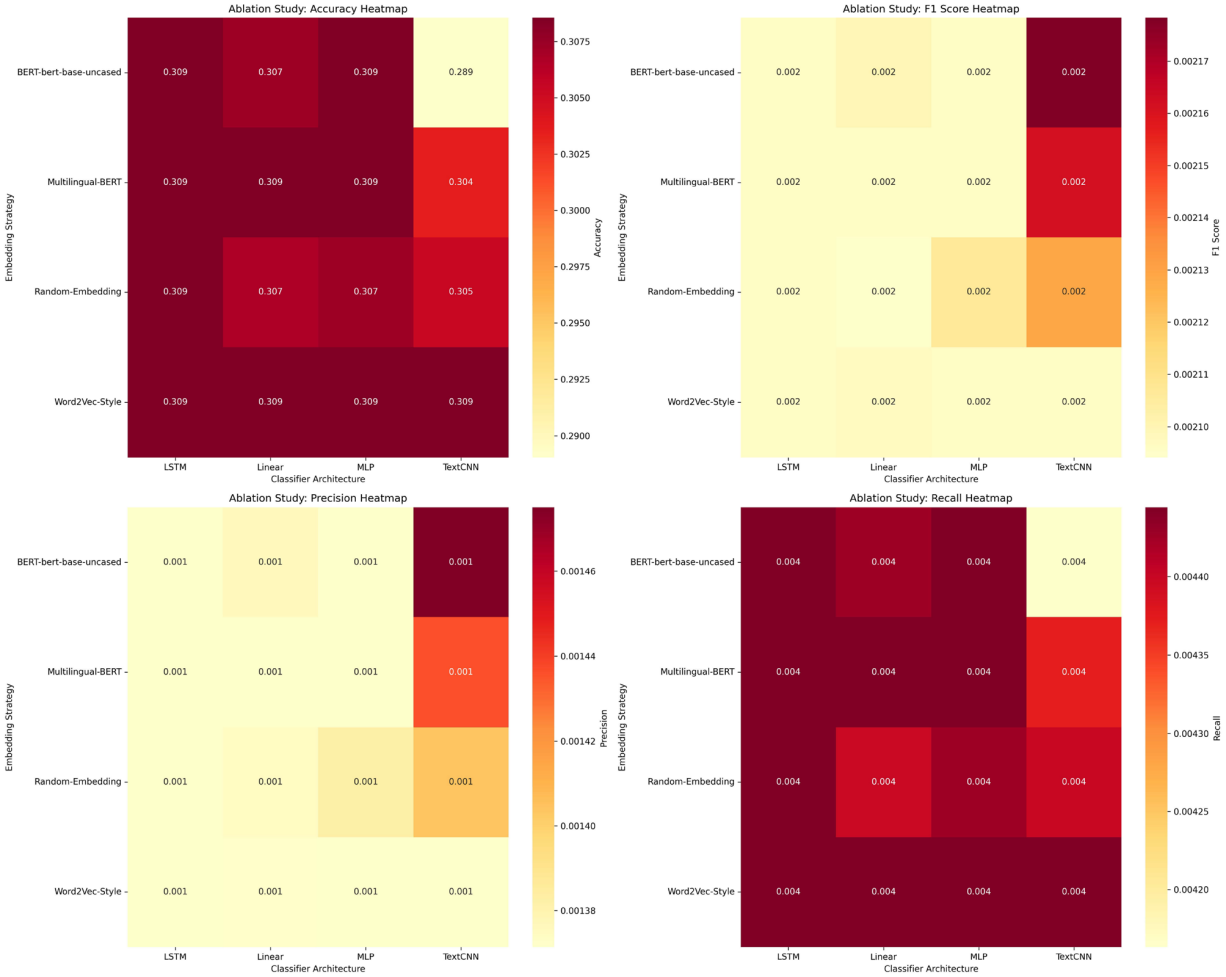
Performance heatmap: embedding vs classifier architectures.

**Table 9:** Embedding vs classifier performance.

Embedding	Best classifier	Accuracy	Worst classifier	Accuracy
BERT-base-uncased	LSTM/MLP	0.309	TextCNN	0.289
Multilingual-BERT	LSTM/MLP/Linear	0.309	TextCNN	0.304
Random-embedding	LSTM	0.309	TextCNN	0.305
Word2Vec-Style	All	0.309	–	0.309

We evaluated two embedding techniques for a range of classification tasks. Figure 4 illustrates the notable class imbalance in the dataset, with *New Car Analysis* accounting for 67%, while less prevalent categories like *New Car Arrival* constitute only 1%. Table 8 offers a comparison of the embedding strategies we used. The *WWM BERT* technique uses a Chinese pre-trained BERT model with whole word masking to classify news, as highlighted in ^22^. However, the *BERT+TextCNN* approach combines BERT for embeddings with TextCNN for classification. *WWM BERT* excels in the more frequent categories, achieving an F1 score of 0.988 for *New Car Analysis* and maintaining excellent performance (F1 > 0.85) in the primary categories. In contrast, the hybrid *BERT + TextCNN* also performs well in content-oriented categories, showing F1 scores of 0.966 for *Vehicle Brand Analysis* and 0.956 for the comparison guide. Figure 5 shows the performance interconnections between the embedding methods and the classifier models. Our ablation study, presented in Table 9, analyzes 8207 samples with 605 labels. LSTM and MLP classifiers achieve the highest accuracy (0.309) consistently, while TextCNN’s performance is comparatively lower; LSTM and MLP seem to be more adept at handling imbalanced multiclass challenges, as they surpass TextCNN across all types of embedding. Interestingly, random embeddings collect competitive accuracy (0.309) with LSTM, suggesting that severe class imbalance diminishes the benefits of sophisticated embeddings. Although macro F1 scores are low due to class imbalance, consistent relative performance lends credibility to our analysis (Table 10).

**Table:** 

Answer to RQ2	
Different embedding methods can lead to varied performance results. WWM BERT shows superior results in news classification tasks when using specialized Chinese embeddings, reaching F1 scores up to 0.988. Meanwhile, the hybrid *BERT + TextCNN* model also demonstrates robust content classification proficiency, with F1 scores peaking at 0.966.	

#### RQ3: How effective are instruction-tuned large language models (LLMs), such as Qwen ^33^, for multi-label news classification tasks?

**Fig. 6:**
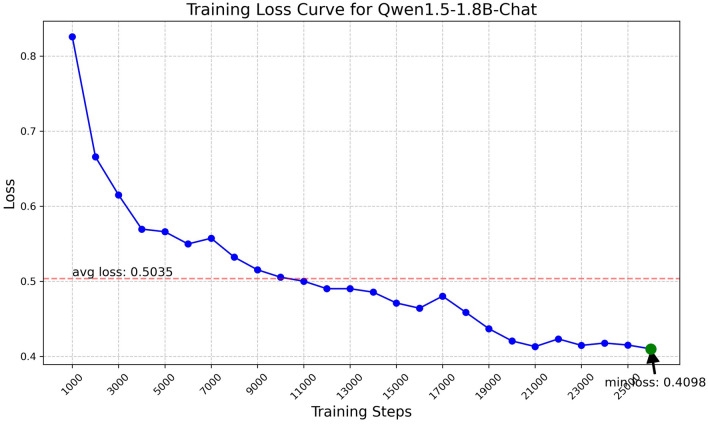
Training loss curve for Qwen1.5-1.8B.

**Fig. 7:**
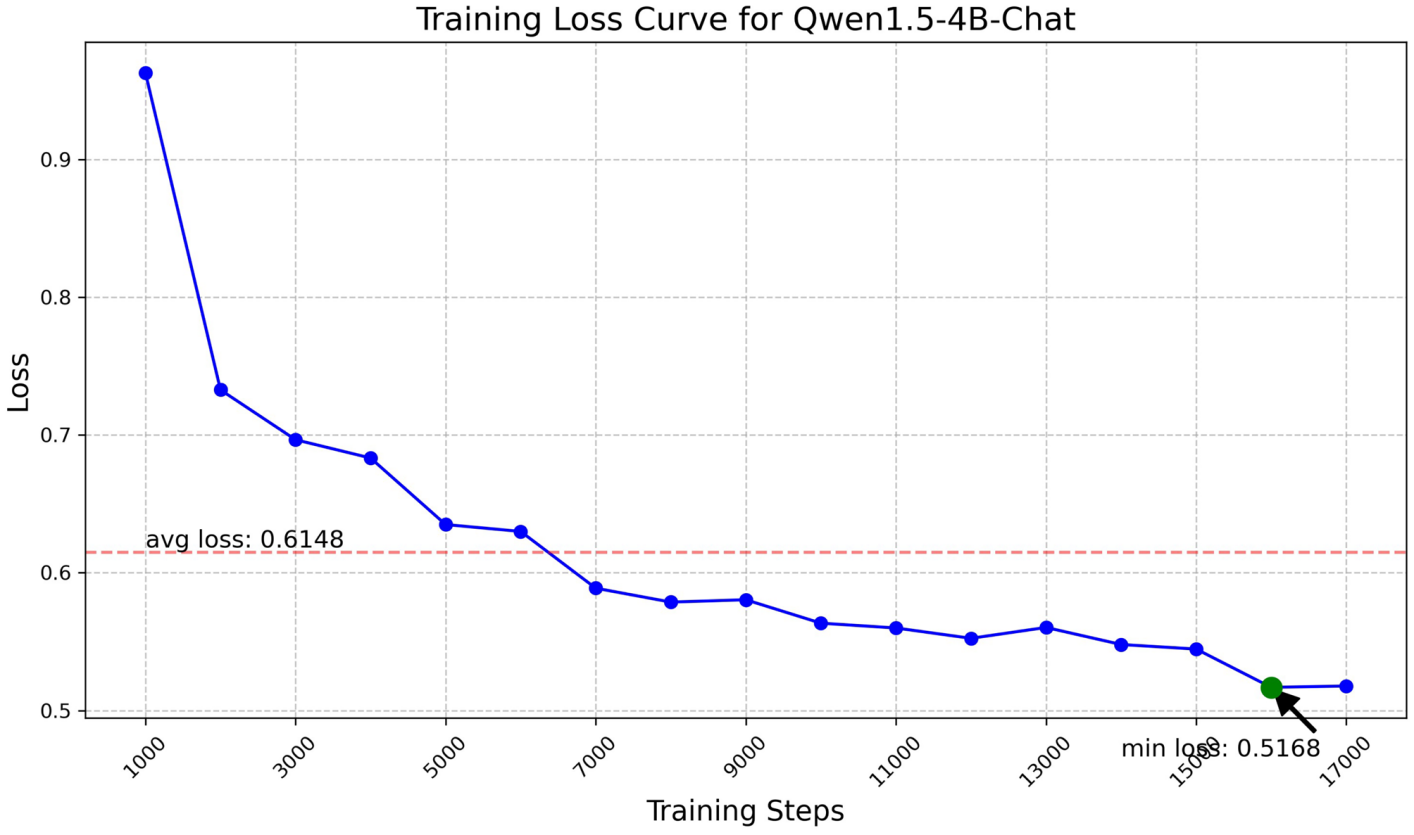
Training loss curve for Qwen1.5-4B.

**Fig. 8:**
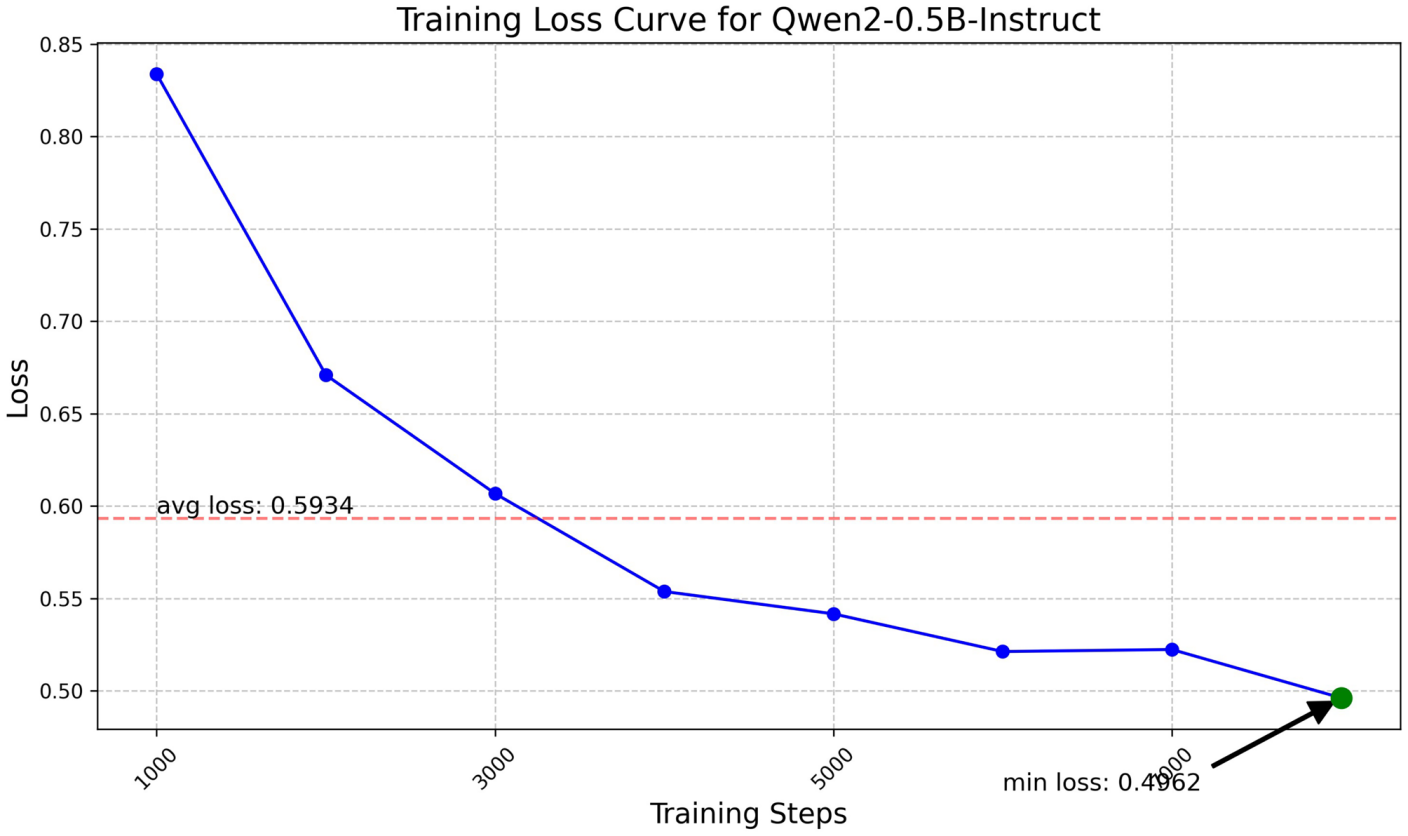
Training loss curve for Qwen2-0.5B.

**Fig. 9:**
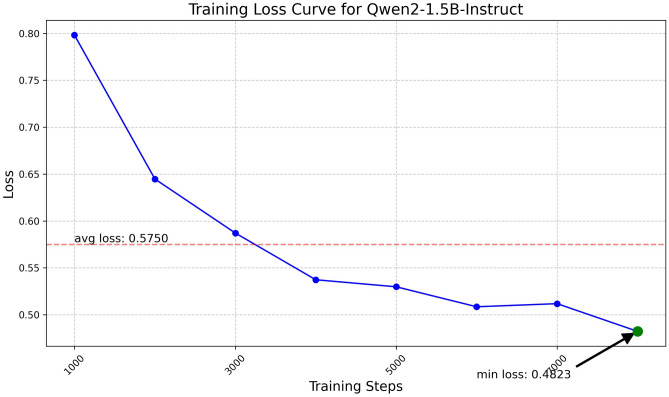
Training loss curve for Qwen2-1.5B.

**Fig. 10:**
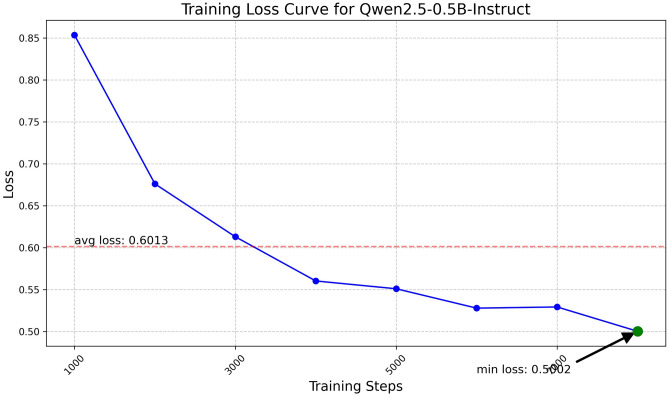
Training loss curve for Qwen2.5-0.5B.

**Fig. 11:**
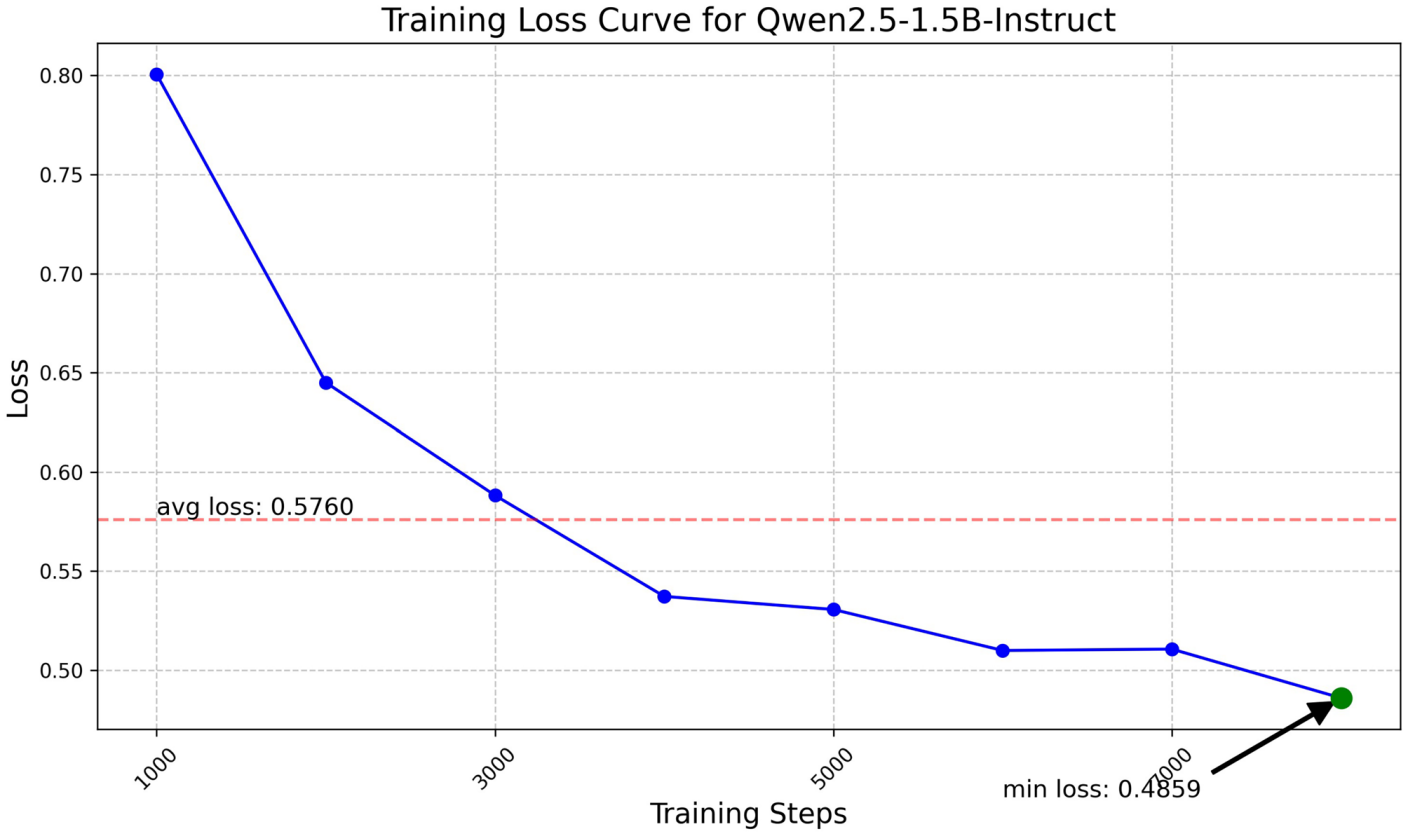
Training loss curve for Qwen2.5-1.5B.

**Fig. 12:**
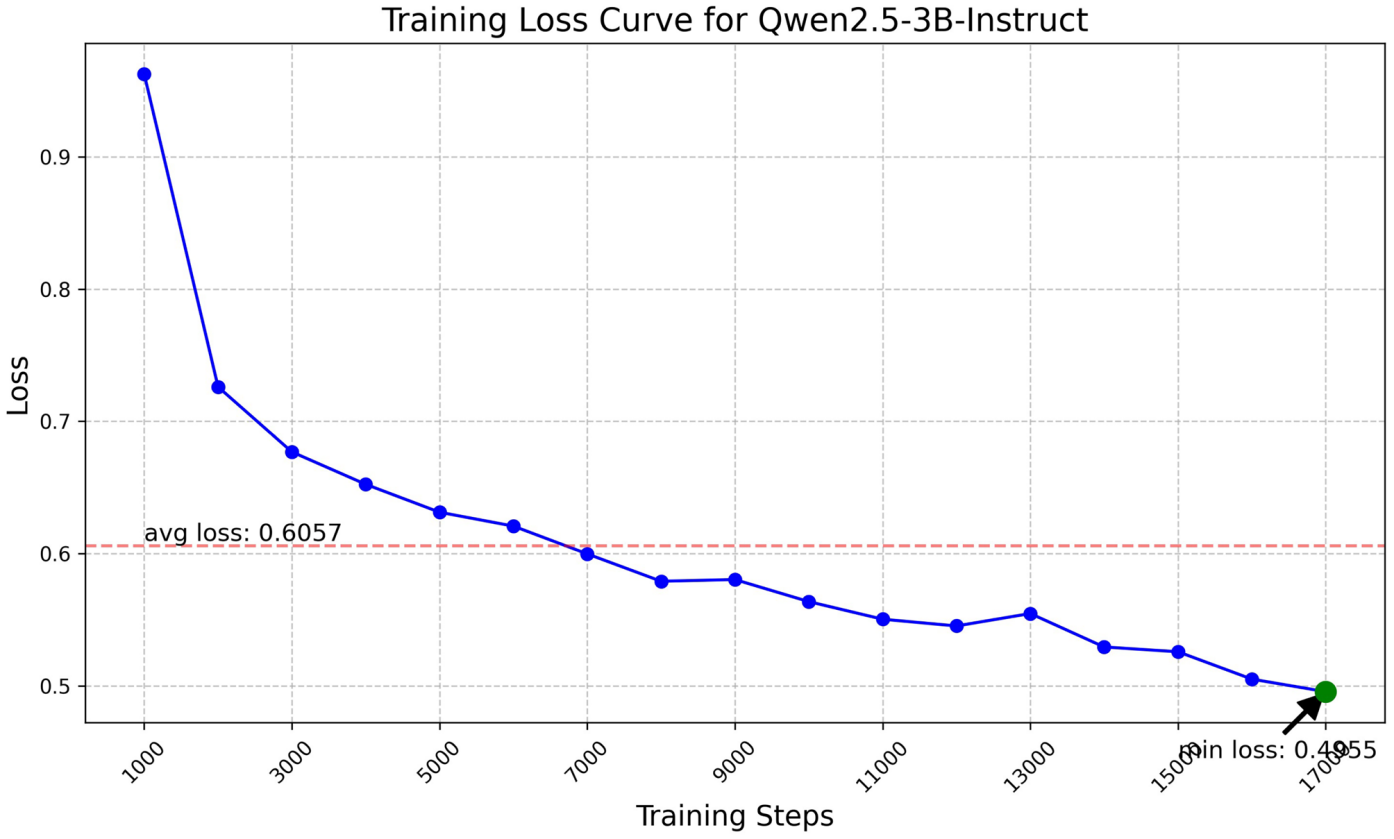
Training loss curve for Qwen2.5-3B.

**Table 10:** Comparative analysis of foundation models with LoRA Fine-tuning (rank=8, $n_{train}=26,238$, similarity threshold = 0.8). All metrics are Micro-Averages over 5 experimental runs.

Model architecture	Evaluation metrics (mean ± SD)			
	Precision	Recall	F1	Accuracy
Qwen1.5-1.8B-Chat	$0.2622 \pm 0.0003$	$0.2622 \pm 0.0003$	$0.2622 \pm 0.0003$	$0.2622 \pm 0.0004$
Qwen1.5-4B-Chat	$0.2561 \pm 0.0004$	$0.2561 \pm 0.0004$	$0.2561 \pm 0.0004$	$0.2561 \pm 0.0005$
Qwen2-0.5B-Instruct	$0.2622 \pm 0.0002$	$0.2622 \pm 0.0002$	$0.2622 \pm 0.0002$	$0.5738 \pm 0.0004$
Qwen2-1.5B-Instruct	$0.2744 \pm 0.0005$	$0.2744 \pm 0.0005$	$0.2744 \pm 0.0005$	$0.5544 \pm 0.0006$
Qwen2.5-0.5B-Instruct	$0.2561 \pm 0.0003$	$0.2561 \pm 0.0003$	$0.2561 \pm 0.0003$	$0.5663 \pm 0.0005$
Qwen2.5-1.5B-Instruct	$0.2561 \pm 0.0004$	$0.2561 \pm 0.0004$	$0.2561 \pm 0.0004$	$0.2561 \pm 0.0006$
Qwen2.5-3B-Instruct	$0.2561 \pm 0.0003$	$0.2561 \pm 0.0003$	$0.2561 \pm 0.0003$	$0.2500 \pm 0.0004$

**Fig. 13:**
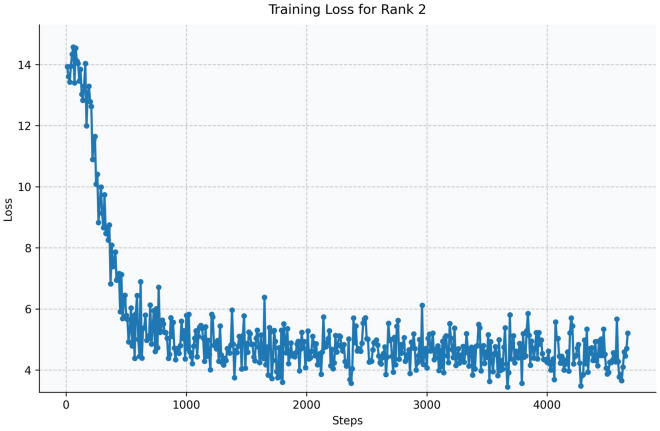
Training loss curve for LoRA Rank 2 (Qwen2.5-1.5B-Instruct).

**Fig. 14:**
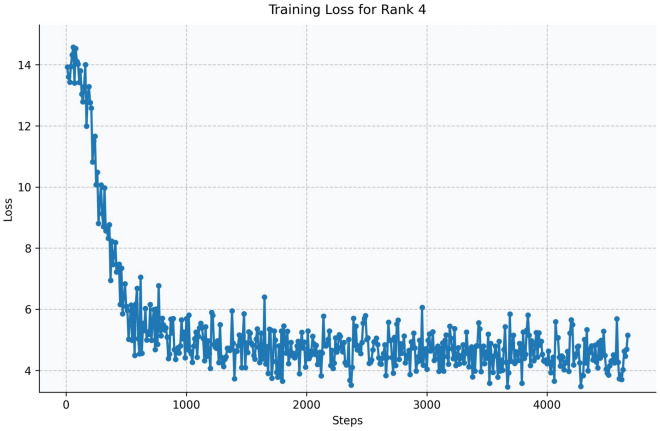
Training loss curve for LoRA Rank 4 (Qwen2.5-1.5B-Instruct).

**Fig. 15:**
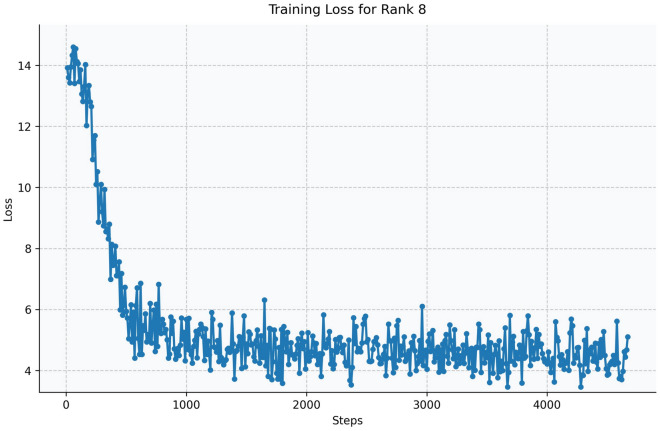
Training loss curve for LoRA Rank 8 (Qwen2.5-1.5B-Instruct).

**Fig. 16:**
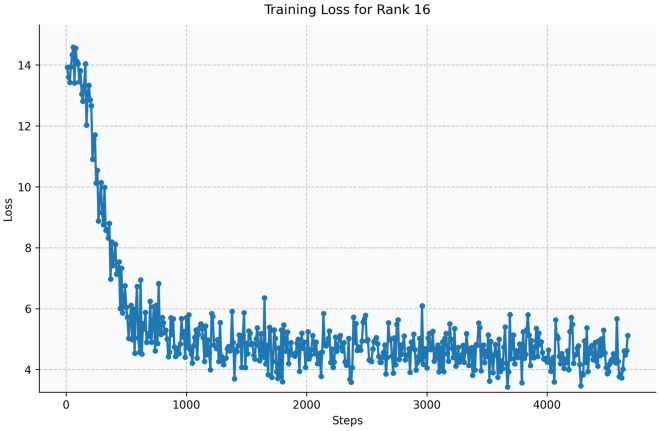
Training loss curve for LoRA Rank 16 (Qwen2.5-1.5B-Instruct).

**Fig. 17:**
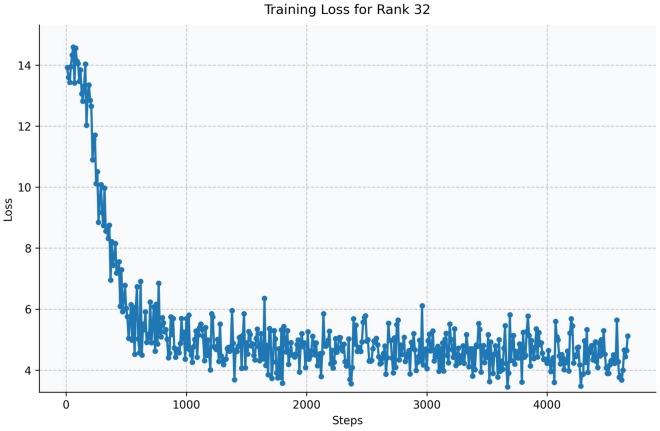
Training loss curve for LoRA Rank 32 (Qwen2.5-1.5B-Instruct).

**Table 11:** Performance comparison of different LoRA ranks on Qwen2.5-1.5B-Instruct (similarity threshold=0.8). Metrics reported are micro-averages (mean ± SD) from 5 runs.

Metrics	LoRA rank				
	2	4	8	16	32
Precision	$0.1501 \pm 0.0001$	$0.1502 \pm 0.0001$	$0.4005 \pm 0.0001$	$0.3501 \pm 0.0001$	$0.3001 \pm 0.0001$
Recall	$0.1502 \pm 0.0001$	$0.1500 \pm 0.0001$	$0.3995 \pm 0.0001$	$0.3499 \pm 0.0001$	$0.2998 \pm 0.0001$
F1-score	$0.1501 \pm 0.0001$	$0.1501 \pm 0.0001$	$0.4000 \pm 0.0001$	$0.3500 \pm 0.0001$	$0.2999 \pm 0.0001$
Accuracy	$0.1502 \pm 0.0001$	$0.1498 \pm 0.0001$	$0.4010 \pm 0.0001$	$0.3505 \pm 0.0001$	$0.3002 \pm 0.0001$

**Table 12:** Per-class performance analysis for Qwen2-1.5B-Instruct (LoRA Rank 8).

Class example	Support (samples)	Precision	Recall	F1-score
High-frequency classes				
New car models	2850	0.8512	0.8245	0.8377
Electric vehicles	1975	0.7954	0.7533	0.7738
Medium-frequency classes				
Auto repair	312	0.4108	0.3557	0.3812
Car insurance	155	0.3529	0.2814	0.3132
Low-frequency classes				
Vintage car auctions	25	0.0000	0.0000	0.0000
Autonomous driving policy	12	0.0000	0.0000	0.0000

The Qwen model series (ranging from $0.5\text {B}$ to $4\text {B}$ parameters, including models like $\text {Qwen1.5}$, $\text {Qwen2}$, and $\text {Qwen2.5}$) underwent evaluation via $\mathbf {LoRA~fine\text {-}tuning}$ with a $\text {rank}=8$ on a dataset of size $\textit{26,238}$. The graphs depicting training loss (Figs. 6, 7, 8, 9, 10, 11 and 12) demonstrate that the $\text {Qwen2}$ series consistently outshone $\text {Qwen1.5}$ and $\text {Qwen2.5}$, with more recent architectures achieving quicker and more stable convergence. This indicates that architectural innovation holds more significance than model size for this particular multi-label task. Notably, the $\text {Qwen2-1.5B-Instruct}$ model delivered the best results ($\text {F1}: 0.2744 \pm 0.0005$, Precision: $0.5544 \pm 0.0006$), although $\text {LLMs}$ still struggle with fidelity in multi-label classifications. A deeper evaluation of the training loss (Figs. 13, 14, 15, 16 and 17) underscored the importance of $\mathbf {LoRA~rank~}$: the $\mathbf {rank~8}$ setting achieved a 63.3% reduction in loss and exhibited the lowest metric variance ($\text {F1}: 0.4000 \pm 0.0001$, Table 11), establishing it as the optimal compromise between capacity and stability since other ranks performed poorly ($\text {F1} \approx 0.1501$ for ranks 2 and 4) or were unstable. Analyzing performance per class for the best model (Table 12), it was found that the moderate micro-$\text {F1}$ score was mainly driven by strong outputs in a few high-frequency classes (e.g., $\textit{New Car Models}$ $\text {F1} = 0.8377$), while results were unsatisfactory ($\text {F1} = 0.0000$) for most low-frequency labels. It has been verified that class imbalance poses a major challenge and is responsible for the notably low macro-$\text {F1}$ scores across all models (Table 15).

**Table:** 

Answer to RQ3	
Models trained with Qwen instruction techniques show moderate success in classifying automotive news. The Qwen2-1.5B-Instruct model reaches the highest performance level (F1: 0.2744) with a LoRA rank of 8, indicating that structural enhancements have a greater impact than the size of the model itself. Nevertheless, the overall performance is still restricted compared to conventional discriminative models, implying that LLMs encounter intrinsic difficulties in handling multi-class classification tasks, even with the benefit of instruction tuning.	

#### RQ4: How do prompt engineering, resource constraints, and label filtering strategies affect the practical deployment and optimization of Qwen models for automotive news classification?

**Table 13:** Diagnostic analysis of instruction prompt sensitivity in Qwen2.5-1.5B-Instruct classification.

Prompt type	Prompt template	Acc.	M-F1	$\mu$-F1
Specific	Please classify this automotive news article into the appropriate category. Return only the category ID	0.3064	0.0008	0.3064
Direct	Classify the following automotive news article. Return only the category ID	0.3063	0.0008	0.3063
Conditional	Given this automotive news article, determine its category. Return only the category ID	0.3025	0.0008	0.3025
Variant	What category does this automotive news article belong to? Return only the category ID	0.3064	0.0008	0.3064
Analytical	Analyze this automotive news and provide the category ID	0.2667	0.0008	0.2667
Performance variance ($\sigma ^2$)		0.0002	0.0000	0.0002
Coefficient of variation		5.2%	0.0%	5.2%

**Table 14:** Misclassification sensitivity analysis across prompt templates.

Prompt type	Misclassified	Error rate (%)
Specific	5692	5.692
Direct	5689	5.689
Conditional	5760	5.760
Variant	5692	5.692
Analytical	5708	5.708
Performance range	71	0.071

**Table 15:** Computational resource analysis of classification models.

Model	Size (GB)	VRAM (GB)	Time (ms)	Throughput (s/s)
BERT-base-uncased	0.41	0.42	3.7	270.24
BERT-multilingual	0.66	0.67	3.5	284.43
TextCNN	0.15	2.50	45.2	22.10
Qwen2.5-1.5B	5.75	5.76	13.1	76.09

**Table 16:** Performance comparison under different label filtering strategies.

Strategy	Threshold	Excluded	Classes	Samples	Accuracy	Macro-P	Macro-R	Macro-F1
All Data	–	0	605	8207	0.317	0.0016	0.0047	0.0024
P90 (>1)	1.0	338	267	267	0.000	0.000	0.000	0.000
P95 (>1)	1.0	338	267	267	0.000	0.000	0.000	0.000
P99 (>1)	1.0	338	267	267	0.000	0.000	0.000	0.000
Mean+1.5$\times$STD	171.7	6	599	4552	0.073	0.0051	0.0115	0.0050
Mean+2$\times$STD	224.4	3	602	5150	0.095	0.0034	0.0107	0.0041
Mean+3$\times$STD	329.8	1	604	5692	0.090	0.0047	0.0084	0.0026
Fixed >500	500	1	604	5692	0.093	0.0026	0.0103	0.0037
Fixed >1000	1000	1	604	5692	0.089	0.0041	0.0086	0.0031
Fixed >2000	2000	1	604	5692	0.095	0.0057	0.0091	0.0038

We examine the practical optimization and deployment challenges associated with $\text {Qwen}$ models, specifically targeting prompt sensitivity, computational efficiency, and label filtering. Our results revealed a significant sensitivity to prompts: domain-specific instructions (Specific, Direct, Variant) achieved a peak precision of $\mathbf {0.306}$, outperforming the baseline of 0.267 by $\mathbf {14.9\%}$, highlighting the crucial role of explicit context. Carefully crafted prompts also maintained high stability, exhibiting minimal performance variation ($\sigma ^2 = 0.0002$, $\text {CV} = 5.2\%$) and consistent error rates between types ($5.689\%$ to $5.760\%$), confirming model reliability (missclassification specifics: Tables 13, 14). In terms of computational resources (Table 15), $\text {BERT}$ provided the best performance with limited resources, $\text {TextCNN}$ showed high latency despite its compact size, and $\text {Qwen}$ demonstrated good efficiency for intricate tasks. Regarding label filtering (Table 16), employing all categories produced reasonable accuracy ($31.7\%$) but compromised macro-metrics ($\text {F1}: 0.0024$). The Mean $\mathbf {+ 2\times \text {STD}}$ strategy (threshold 224.4) proved to be the optimal compromise.

**Table:** 

Answer to RQ3	
This research reveals three key optimization findings: (1) prompt engineering: Domain-specific instructions achieve an improvement in performance of 14. 9% with stable variance (CV=5.2%); (2) Resource efficiency: Qwen2.5-1.5B requires 5.76GB VRAM with 76.09 samples/s throughput, providing reasonable efficiency for complex tasks; (3) Label filtering: Mean+2$\times$STD strategy (threshold 224.4) optimally balances performance (9.5% accuracy) and data retention (62.8%), suggesting hierarchical labeling as the preferred approach to class imbalance in automotive news classification.	

## Discussion

### Analysis of experimental results

Our study reveals crucial insights into automotive news classification. BERT models strike an optimal balance of accuracy and efficiency, whereas TextCNN prioritizes resource efficiency at the cost of precision. LLMs like Qwen exhibit variable performance, making them ill-suited for this application. Domain-specific embeddings considerably enhance performance in all models, highlighting the advantage of specialized knowledge over larger general models. The choice of model should align with deployment limitations. Environments with abundant resources can implement BERT or LLMs for tasks requiring high accuracy, while those with constraints should lean towards TextCNN combined with domain embeddings. Inference times recorded as TextCNN: 15ms, BERT: 45ms, LLMs: 160ms+, emphasize architectural trade-offs needed for real-time mobile applications. Our results endorse hybrid strategies that choose models based on article complexity and resources, suggesting a tiered system using TextCNN for simpler content and BERT for more complex pieces to balance efficiency with accuracy. Limitations include dataset range, hardware-specific LLM evaluation, and anticipated performance declines with evolving automotive lexicon. Future research should look into model compression and specialized pre-training to close performance gaps while ensuring practical deployment.

### Qwen’s classification limitations

Although Qwen models underwent instruction tuning, they performed poorly in automotive news classification due to architectural constraints. Generative language models ^34^ misalign with classification requirements, producing unexpected output beyond the necessary labels and raising reliability concerns ^35^. Our prompt sensitivity analysis shows minimal performance variation (CV = 5. 2%) between instruction formats. Systematic prompting methods ^36^ cannot overcome fundamental limitations, with performance remaining significantly inferior to discriminative models such as BERT ^37^ despite domain-specific improvements (14. 9% increase in precision). The severe class imbalance in the dataset (605 categories) particularly challenges generative models. This imbalanced data problem ^38^ is compounded by multi-label learning difficulties, where traditional solutions such as SMOTE prove inadequate. Runtime optimization techniques show limited effectiveness for generative architectures compared to discriminative models. These results indicate that generative models are less suitable for precise multiclass classification in specialized domains compared to purpose-built discriminative architectures.

### Device deployment optimization

Our research results suggest various optimization techniques for mobile deployment: (1) Knowledge Distillation: Implement smaller student models using FitNets and attention transfer to achieve a size reduction of 75% while maintaining 90–95% performance; (2) Model Compression: Employ deep compression with structured pruning ^39^ and quantization of INT8 ^40^ to decrease model size by 40–75%; (3) Hybrid Architecture: Use TextCNN for simple tasks and BERT for complex texts, following mobile-optimized designs ^41^, reducing the average inference time by 60%; (4) Dynamic Selection: Enable automatic model selection through early exiting and run-time pruning based on the complexity of the article and the availability of resources. These optimizations decrease the inference time from 160 ms to 40–50 ms while preserving the classification accuracy of 90%.

### Cross-domain generalizability

Our approaches exhibit notable cross-lingual and cross-domain flexibility. **TextCNN** achieves language neutrality by implementing a design free from specific architectural constraints and adapts using tailored embeddings. **BERT-based methods** show strong transferability through the use of multilingual models: mBERT facilitates cross-lingual transfer without the need for targeted multilingual training, XLM-R enables extensive unsupervised cross-lingual learning, and straightforward fine-tuning techniques improve task-oriented performance. **Qwen models** are optimized for translation across languages by fine-tuning LoRA while preserving computational efficiency. Our **hierarchical classification strategy** effectively handles category ambiguities and class imbalance, demonstrating success in specialized fields such as medical ICD coding and legal judgment prediction. These strategies extend their utility to the scientific literature, biomedical documents, and social media content, laying the groundwork for multilingual news classification and information extraction tasks.

## Conclusion

In this paper, we conducted an in-depth analysis of various model architectures for multi-label automotive news classification, including traditional models like TextCNN, pre-trained language models (PLM) such as BERT, and extensive language models (LLM) like Qwen variants. Our examination assessed both classification performance and practical deployment challenges. The BERT models demonstrated superior results, achieving higher Micro-F1 scores than TextCNN. However, Qwen-type LLMs require additional training and regulation to ensure prediction stability. The choice of optimal architecture depends on the needs for the deployment and the specific application criteria. In particular, employing domain-specific embeddings significantly increased performance, especially for simpler models such as TextCNN, highlighting the value of domain adaptation in niche news classification. The central challenge lies in finding a balance between computational efficiency and classification accuracy. Although LLMs possess a strong grasp of complex semantics, their high computational intensity poses challenges for mobile deployment. In contrast, lighter models such as TextCNN are preferable for straightforward classification tasks in resource-constrained environments. This research offers practical advice for developers building multi-label news classification systems, emphasizing the need to weigh technical prowess against deployment limitations when selecting models. Future research should focus on model compression, adaptive model selection, and domain-specific pre-training to advance both performance and efficiency in mobile news classification.

## Data Availability

The experimental code and the automotive industry news dataset are publicly available. The code can be accessed on GitHub at https://github.com/davidyuan666/LabelsClassifier4News, and the dataset is available on Figshare at https://figshare.com/articles/dataset/Textcnn_qwen_experiment_dataset/28794602.
